# Microcoulomb (0.7 ± $\frac{0.4}{0.2}$ μC) laser plasma accelerator on OMEGA EP

**DOI:** 10.1038/s41598-021-86523-5

**Published:** 2021-04-05

**Authors:** J. L. Shaw, M. A. Romo-Gonzalez, N. Lemos, P. M. King, G. Bruhaug, K. G. Miller, C. Dorrer, B. Kruschwitz, L. Waxer, G. J. Williams, M. V. Ambat, M. M. McKie, M. D. Sinclair, W. B. Mori, C. Joshi, Hui Chen, J. P. Palastro, F. Albert, D. H. Froula

**Affiliations:** 1 Laboratory for Laser Energetics, University of Rochester, Rochester, NY 14623 USA; 2 California State University Stanislaus, Turlock, CA 95382 USA; 3 Lawrence Livermore National Laboratory, Livermore, CA 94550 USA; 4 University of Texas at Austin, Austin, TX 78705 USA; 5 University of California Los Angeles, Los Angeles, CA 90095 USA

**Keywords:** Laboratory astrophysics, Plasma-based accelerators, Ultrafast lasers

## Abstract

Laser-plasma accelerators (LPAs) driven by picosecond-scale, kilojoule-class lasers can generate particle beams and x-ray sources that could be utilized in experiments driven by multi-kilojoule, high-energy-density science (HEDS) drivers such as the OMEGA laser at the Laboratory for Laser Energetics (LLE) or the National Ignition Facility at Lawrence Livermore National Laboratory. This paper reports on the development of the first LPA driven by a short-pulse, kilojoule-class laser (OMEGA EP) connected to a multi-kilojoule HEDS driver (OMEGA). In experiments, electron beams were produced with electron energies greater than 200 MeV, divergences as low as 32 mrad, charge greater than 700 nC, and conversion efficiencies from laser energy to electron energy up to 11%. The electron beam charge scales with both the normalized vector potential and plasma density. These electron beams show promise as a method to generate MeV-class radiography sources and improved-flux broadband x-ray sources at HEDS drivers.

## Introduction

**Figure 1:**
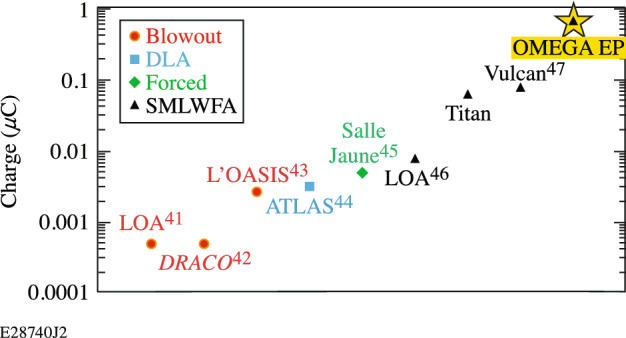
Plot of the maximum charge of the electron beams produced by laser-plasma accelerators at different facilities ^41– 47^. The yellow star is the result reported in this work.

Laser-plasma accelerators (LPAs) driven by short-pulse, kilojoule (kJ)-class lasers provide a path to producing compact sources of high-charge, high-energy electron beams for conversion into x-ray and positron sources. These sources could be readily coupled to multi-kilojoule, high-energy–density science (HEDS) drivers including the OMEGA laser at the Laboratory for Laser Energetics, the National Ignition Facility at Lawrence Livermore National Laboratory, the Laser Mégajoule at Commissariat à l'Energie Atomique, and the Z Machine at Sandia National Laboratory, due to their proximity to short-pulse, kJ-class lasers (OMEGA EP, Advanced Radiography Capability, Petawatt Aquitaine Laser, and Z Beamlet, respectively). Continued improvement of x-ray sources coupled to HEDS drivers is a constant priority at these facilities for backlighting and radiography. LPA-based x-ray sources utilizing self-modulated laser wakefield acceleration (SMLWFA) have already shown promise as MeV-class radiography or high-flux broadband sources^1–10^. SMLWFA-based sources can deliver such high fluxes on account of the two orders of magnitude higher electron charge that they deliver compared to other LPA sources (Fig. 1), and the x-ray yield from all three mechanisms (betatron, bremsstrahlung, and inverse Compton scattering) used to convert electrons to x-rays increase with the number of electrons in the electron beam.

A SMLWFA-based LPA can accelerate significantly more charge than a laser wakefield accelerator (LWFA) driven by an ultrashort laser (pulse duration τ < plasma wavelength λ_p_), where charge is only trapped in a few plasma periods. In SMLWFA, a laser pulse with τ > λ_p_ enters a plasma. The front portion of that pulse is reflected back as a lower-frequency wave in a process known as stimulated Raman backscatter^11^. The laser pulse becomes modulated at the plasma wavelength via the Raman forward scattering^12,13^ and/or self-modulation^14–17^ instabilities. These modulations lead to a train of laser micropulses coherently driving plasma waves whose longitudinal electric field can trap and accelerate electrons to relativistic energies.

Simulations show that for the laser and plasma parameters explored in the experimental work presented here, the electrons are accelerated by both the longitudinal wakefield and direct laser acceleration (DLA)^1^. In any LWFA, the electrons accelerating in the plasma waves undergo betatron oscillations about the laser axis due to the restoring force of the ion column that forms behind the drive laser. If, as they oscillate, the electrons overlap with the laser, their betatron motion can be enhanced by the transverse laser field. The magnetic field of the laser, through the **v** × **B** force, then continuously converts the transverse momentum of the electrons into longitudinal momentum in a process referred to as DLA^18–28^.

Here, we report on the first LPA driven by a short-pulse, kJ-class laser (OMEGA EP) connected to a multi-kilojoule HEDS driver (OMEGA). The produced electron beams have maximum energies that exceed 200 MeV, divergences as low as 32 mrad, record-setting bunch charges exceeding 700 nC, and laser-to-electron conversion efficiencies up to 11%. The bunch charge is comparable to high-bunch-charge radio-frequency (rf) accelerators (~ 1 μC), but with sub-picosecond durations versus the millisecond durations characteristic of rf sources. These electron beams are, to our knowledge, the highest-charge and highest-conversion-efficiency electron beams produced from an LPA.

## Experimental setup

**Figure 2:**
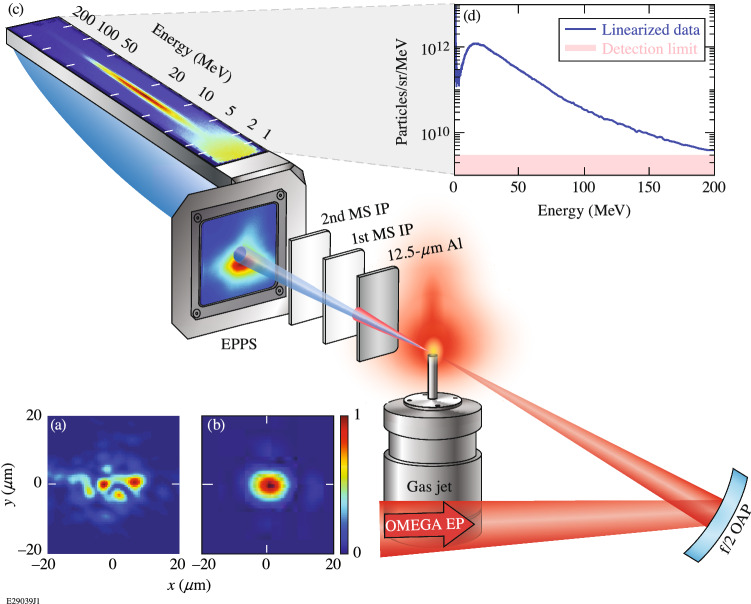
(**a**,**b**) Examples of target spot at focal plane for the standard OMEGA EP f/2 focus and the f/6 apodized focus, respectively, measured on-shot using a hybrid Shack-Hartmann-phase-retrieval-based wavefront sensing method^32,33^. The peak fluence per energy for (**a**) and (**b**) is 7.9 and 11.8 × 10^5^ cm^−2^, respectively. Note that the apodized focal spot in (**b**) produces a single, high-intensity laser spot ideal for driving high-quality SMLWFA in contrast with the multiple hot spots that exist with the f/2 focus. (**c**) Relative layout of the laser, target, and diagnostics. (**d**) Electron spectrum from an a_0_ = 5.1 laser shot propagating through a plasma density of 5.4 × 10^18^ cm^−3^ generated by a 6-mm-diameter nozzle. The shaded region marks the detection limit of the EPPS. OAP: off-axis parabola.

Experiments were performed on the OMEGA EP Laser System^29^ at LLE. The laser was run with a central wavelength λ of 1054 nm at best compression (pulse duration of 700 ± 100 fs). To improve the quality of the focal spot and increase the Rayleigh length, the focusing geometry of the short-pulse laser beams was converted from its nominal f/2 geometry by using spatially filtered apodizers^30^ located at the injection plane before amplification in the Nd:glass beamline to control the beam diameter and generate an f/5, f/6, f/8, or f/10 geometry. The properties of these configurations are summarized in Table 1, and nominal focal spots at the target plane for the standard f/2 focus and the f/6 apodizer are shown in Fig. 2a,b, respectively. Note that no electrons with energies exceeding 14 MeV were produced in shots where the f/2 configuration was fielded. At focus, the R80 spot size of the laser (i.e., radius that contains 80% of the total energy) was between 11.5 and 19.9 μm. The apodized laser energy varied from 10 to 115 J, which produced on-target peak normalized vector potentials (a_0_ $\cong 8.6 \times {10}^{-10}\sqrt{{\mathrm{I}}_{0}\left[\mathrm{W}/{\mathrm{cm}}^{2}\right]}\uplambda \left[{\upmu \text{m}}\right]$, where I_0_ is the vacuum intensity) between 1.8 and 6.7. The apodized laser pulse was focused 500 μm inside a Mach 5 gas jet with nozzle diameters varying between 2 and 10 mm as shown in Fig. 2c. The gas was 100% He, and the resultant plasma densities in the plateau ranged from 1.5 × 10^18^ to 4.5 × 10^19^ cm^−3^ depending on nozzle diameter and backing pressure. The gas jet was an ultrafast (opens and closes in ~ 100 μs) system specifically designed to limit the total gas release in the event of failure in order to protect the sensitive electronics in the compressor^31^.

**Table 1:** Properties of injection-plane apodizers used for this work.

Configuration	Average R80 [μm]	Max. energy [J]	Max. a_0_	Peak intensity [W/cm^2^]
f/5	14.8 ± 1.7/1.3	135	6.6	5.3 × 10^19^
f/6	14.0 ± 3.0/2.5	85	6.7	5.5 × 10^19^
f/8	16.2 ± 3.7/2.0	55	3.9	1.8 × 10^19^
f/10	18.1 ± 0.6/0.3	40	2.3	6.5 × 10^18^

The maximum energy is given for operation at best compression, i.e. duration of 700 ± 100 fs. The maximum a_0_ value given in column four and the peak intensity given in column five are the maximum values calculated from the laser energy, spot size, and pulse duration measured during this course of experiments.

## Results and discussion

**Figure 3:**
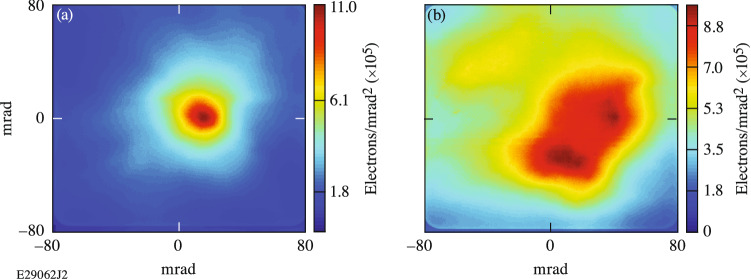
Plot of transverse electron beam profiles for (**a**) the lowest-divergence and (**b**) the highest-charge electron beams. Both laser shots were taken with an f/5 apodizer.

Figure 3a shows the transverse profile of the lowest-divergence electron beam produced in this experiment. The charge in this beam was 148 nC and was produced by an a_0_ = 4.4 laser shot propagating through a plasma density of 1.1 × 10^19^ cm^−3^ generated by a 10-mm-diameter nozzle. The divergence of this beam was 32 × 39 mrad, and it was pointed 8 mrad from the axis of the electron–positron–proton spectrometer (EPPS). The divergence was calculated by fitting the lineout of the transverse profile through the peak of the electron beam with a Lorentzian and taking the full width at half maximum (FWHM). The total charge in the FWHM was 19 nC.

This divergence demonstrates a major step forward in the possible quality of electron beams from SMLWFA since it is significantly reduced from the next best divergence reported from other SMLWFA experiments (64 × 100 mrad ± 10 mrad^6^) and is on the order of the divergences (< 10 mrad) of the electron beams produced by LWFA being driven by ultrashort-pulse lasers (τ < λ_p_). The possibility of lower-divergence electron beams from SMLWFA-based LPAs increases their utility when using the produced electron beams to generate compact sources of high-energy electrons for conversion to photons and positrons. The increased directionality of these electron beams makes them easier to transport and to point either to converter targets or to the interaction being probed. Lower-divergence electron beams will mean a lower source size, and therefore a higher spatial resolution, for bremsstrahlung or inverse Compton scattering x-ray sources generated using these electron beams. For the lowest-divergence electron beam reported here, that source size would be approximately half of that produced by the electron beam from Ref.^6^. Lower-divergence electron beams also reduce the background noise in these applications.

The nature of SMLWFA means that there is variation in the reproducibility of the electron beam quality. The divergence of the electron beams will be affected by the plasma density profile and uniformity, the laser focal spot quality and size, the phase front of the laser, and the interaction between the laser and the plasma, including the coupling of the laser into the plasma and the subsequent laser evolution (modulation and self-focusing). For all but three of the 23 high-plasma-density (n_e_ > 1.9 × 10^19^ cm^−3^) shots in this experiment, the produced electrons did not form a defined beam, and instead, the transverse charge profile was distributed across the entire solid angle collected by the EPPS. For those three shots, all were produced in 10-mm nozzles, which suggests that having longer plasmas, and therefore longer distances for laser evolution, may help maintain the transverse beam profile. For the remainder of the 49 shots with charge ≥ 50 nC, the shots were either single-peaked with higher divergence than the shot shown in Fig. 3a or had multiple peaks.

Figure 3b shows the electron-beam profile for the highest-total-charge (707 nC) electron beam, which has a much larger divergence than the lowest-divergence shot shown in Fig. 3a and two distinct charge peaks. This electron beam was produced by an a_0_ = 6.6 laser shot propagating in a plasma density of 7.5 × 10^18^ cm^−3^ created by a 6-mm-diameter nozzle. Although this highest-charge electron beam shows that there was variability in the transverse beam quality in the LPA platform being developed here, it is still on the order of the best divergences reported in other SMLWFA experiments, while still having at least a factor of 10 more charge. 50% of the total charge is encompassed in a 53.9 mrad and 59.4 mrad radius for the beam profiles shown in Fig. 3a,b, respectively.

**Figure 4:**
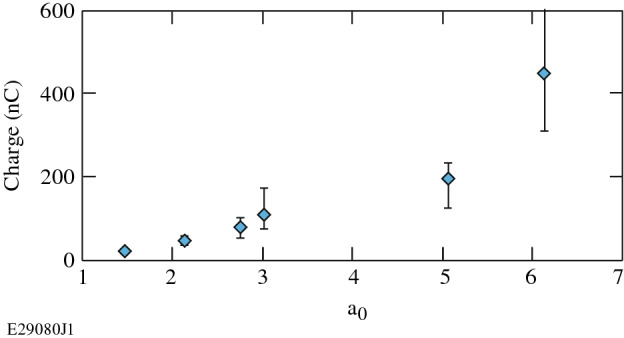
Electron beam charge versus a_0_ for a 6-mm-diameter nozzle operating at a plasma density of 5 × 10^18^ cm^−3^.

Figure 4 shows that the total charge in the electron beams scales approximately linearly with a_0_. The data shown is for a 6-mm-diameter nozzle operating at a plasma density of 5 × 10^18^ cm^−3^, but plasma densities of 1, 2, and 3 × 10^19^ cm^−3^ showed the same trend. This trend was also seen for 4-mm-diameter nozzles operating at 1 × 10^19^ cm^−3^ and 10-mm-diameter nozzles at densities of 0.2, 0.5, 1, and 3.5 × 10^19^ cm^−3^. There are several potential factors affecting this observed scaling. Quasi-3D^34^ OSIRIS simulations of one of the shots from this experiment (a_0_ = 6, n_e_ = 7.5 × 10^18^ cm^−3^, nozzle diameter = 6 mm) show that for this parameter regime, the laser begins to pump deplete as early as 1 mm into the constant-plasma-density region. In this experiment, because the spot size is approximately constant, a higher a_0_ is correlated to higher laser energy, and therefore the pump depletion may be mitigated. A higher a_0_ is also correlated to a higher laser power, which means that a larger percentage of the 700 fs pulse duration will have a power higher than the critical power for self-focusing^35^. This means that a larger percentage of the laser pulse will not be diffracted, which also mitigates pump depletion, and can contribute to the series of laser micropulses driving the wake, and thus produce more plasma periods accelerating charge. Higher a_0_ values are also associated with longer plasma periods in the wakefield^36,37^, which can hold more charge. The charge in the electron beams was calculated using the method described in “Methods”.

**Figure 5:**
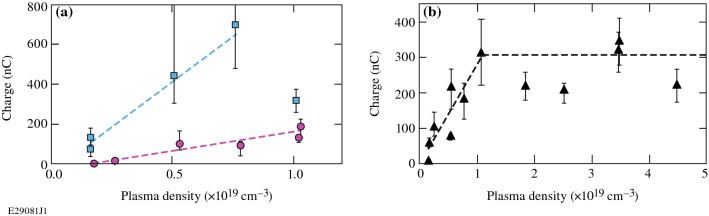
Electron beam charge as a function of plasma density (**a**) up to ~ 1 × 10^19^ cm^−3^ for a_0_ ~ 3 (magenta circles) and a_0_ $\gtrsim$ 6 (blue squares) for a 6-mm-diameter nozzle and (**b**) over the entire sampled plasma density range for a_0_ ~ 5 and a 10-mm-diameter nozzle. The dashed lines are added to guide the eye.

Figure 5a shows that the charge in the electron beam scales approximately linearly with plasma density until a density of 1 × 10^19^ cm^−3^. The two data sets shown each have a different a_0_ value; the rate of increase of charge with plasma density is steeper for the higher a_0_ value. The highest-charge electron beam measured in this experiment, which had a charge of 707 ± 429/224 nC, was produced at an a_0_ of 6.6 and a plasma density of 7.5 × 10^18^ cm^−3^. Using an electron energy of 17.9 MeV, which is the weighted average electron energy of the representative electron spectrum from this experiment (Fig. 2d), this charge corresponds to a conversion efficiency from laser energy to electron energy of 11%. The details of this calculation are included in “Methods”. 30%, 50%, and 90% of that total energy is contained in electrons with energies below 18.5 MeV, 25.6 MeV, and 85.1 MeV, respectively.

In this experiment, trapping was observed to begin at a plasma density of 1.5 × 10^18^ cm^−3^. About 30% of the shots taken at this density produced measurable charge. Above a plasma density of 2.4 × 10^18^ cm^−3^, charge was trapped on every shot. Measurable charge was first observed for P/P_crit_ values of 3.4, where P is the laser power and P_crit_ is the critical power required for relativistic self-focusing. This value is in reasonable agreement with the P/P_crit_ ~ 3 threshold measured for LWFA in the blowout regime^38^. Measurable charge was seen in 1/3 of shots at this P/P_crit_ value. Charge was consistently trapped once P/P_crit_ exceeded 5.2. Figure 5b shows that when the charge scaling is extended to higher plasma densities, the maximum charge produced plateaus with density. A similar trend was seen for data taken on a 6-mm-diameter nozzle for both a_0_ values of 5 and 6.

## Conclusions

A microcoulomb-class, high-conversion-efficiency laser-plasma accelerator was demonstrated, providing the first laser-plasma accelerator driven by a short-pulse, kJ-class laser (OMEGA EP) connected to a multi-kJ HEDS driver (OMEGA). The produced electron beams have maximum energies that exceed 200 MeV, divergences as low as 32 mrad, record-setting charges that exceed 700 nC, and laser-to-electron conversion efficiencies up to 11%. Total charge in the electron beam is found to scale with both a_0_ and plasma density. Based on these empirical scalings, higher-charge electron beams may be possible using laser systems that can deliver a_0_ values larger than the maximum a_0_ of 6.7 produced in this configuration while still maintaining longer f/#s and near-Gaussian, single-moded laser spots on target. These electron beams are, to our knowledge, the highest-charge electron beams produced from a laser-plasma accelerator and are well poised as a path to MeV-class radiography sources and improved flux for broadband sources of interest at HEDS facilities.

## Methods

### Experimental setup

In the OMEGA EP experimental chamber, the produced electron beam propagated 47.63 or 56.52 cm downstream where it was intercepted by an Electron Positron Proton Spectrometer (EPPS), which was mounted using a ten-inch-manipulator, with a modified blast shield on the front. This modified blast shield held an image-plate (IP) stack orthogonal to the electron beam for measurements of the transverse electron beam profile, the divergence, the electron-beam pointing, and charge. The image plate stack on the front of the EPPS consisted of 12.5 μm or 25 μm of aluminum, to block transmitted laser light, followed by two FujiFilm BAS-MS image plates. These image plates will be referred to as the first and second “front” image plates for the remainder of this paper. The first front image plate acted as a filter for electrons with energies < 400 keV. Any x-rays produced either on the foil or in the wakefield itself were negligible compared to the signal produced by the electrons on the IPs. The second front image plate recorded the transverse profile, the divergence, the pointing, and the charge of those electrons that were energetic enough to pass through the first front image plate. No charge was recorded on the second front image plate when the laser was fired with no gas target. The front image plates were run with or without a hole at the center; the hole was used to allow the electrons to propagate unaffected into the pinhole of the spectrometer portion of the EPPS and be dispersed. The spectrometer portion of EPPS was operated with the high-energy/low-dispersion magnet pack and therefore has a maximum energy resolution of 200 ± 20 MeV, so any electrons with energies exceeding 200 MeV were not resolved. In this experiment, electron beams with energies up to this maximum resolvable energy were measured.

### Charge measurement

The photostimulated luminescence (PSL) signal from the second front image plate of the image plate stack on the front of the EPPS was used to measure the charge of the incident electron beam. When an electron of a known energy is incident on an image plate, the response of that image plate in photostimulated luminescence (PSL) is known^39,40^ as shown in Figure 7 of Ref.^39^. Thus, for a monoenergetic electron beam, it is straightforward to scan an image plate, integrate the total number of PSL from that image plate, and then convert that PSL to charge using the known response (PSL/electron). In this experiment, this process was used except that a weighted PSL/electron conversion factor, calculated based on the measured electron spectrum, was used to account for the fact that the incident electron beams are not monoenergetic. We calculate this weighted PSL/electron conversion factor by taking a representative electron spectrum from the experiment (Fig. 2d), integrating along energy from 0.9 to 200 MeV (the range resolvable by the EPPS) to find the total number of particles/sr in that spectrum, and then calculating what percentage of the total number of particles/sr are at each energy. The percentage at a given electron energy is then multiplied by the PSL/electron response at that energy, and the products of each multiplication are summed to produce a single conversion factor for the electron spectrum. For this case, that weighted conversion factor was 0.026 PSL/electron. Note that this method of calculating the weighted conversion factor assumes that the electron spectrum is constant over the entire divergence. Once this weighted conversion factor was determined, we could simply sum the total PSL measured on the second front image plate and convert the total PSL to charge. In this calculation, the total measured charge reported was determined within the solid angle of the front image plates. Note that for the highest-charge shot only, the EPPS detector was located at a distance of 47.63 cm from target chamber center, and so the reported charge is over a solid angle of 26 msr, unlike the remainder of the shots, where the EPPS sat at 56.52 cm from target chamber center, and so the reported charge is over a solid angle of 18 msr. The charge in the highest-charge shot contained in the 18 msr aperture is 600 ± 185/162 nC. For those image plates where a hole was present, a Gaussian fit to the data was used to estimate the charge that passed through the hole. The error introduced by making this correction is included in the error bars.

**Figure 6:**
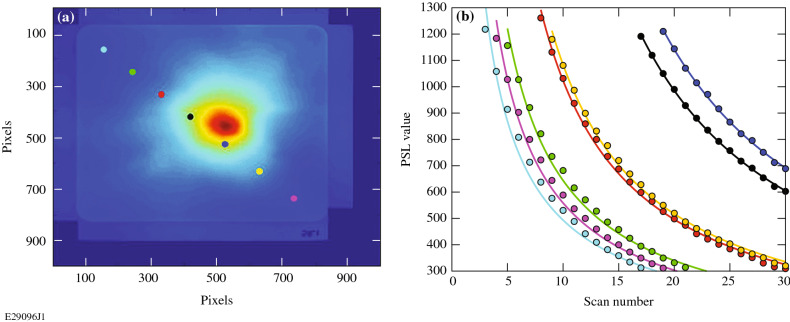
(**a**) Front image plate after being scanned 30 times to remove all saturation in the readout. Colored points mark the seven locations where the decay of the PSL value with scan number is recorded. (**b**) PSL values versus scan number at the seven locations. Colors of the curves correspond to the colors of the points in (**a**) across the image plate. Solid lines are the power fits to each curve.

Due to the significant amount of charge generated in this experiment, part or all of the readout of the second front image plate was saturated. Because the PSL signal decreases by a prescribed amount each time an image plate is scanned, the front image plates were scanned repeatedly until no saturation in the readout existed. The measurement was recorded for seven locations shown in Fig. 6a distributed diagonally across the second front image plate after each scan. As seen in Fig. 6b, the decay of the PSL signal with scan number for each of the seven points takes the form of a power distribution PSL = αN^β^, where PSL is the signal and N is the number of scans of the image plate. The decay of the PSL signal for each of the seven points was fitted with the power distribution to recover the fit parameters α and β for each point. Those fit parameters from the seven points were averaged to produce an average decay of the signal on the image plate for each scan. The total signal from image plates that have a saturated readout on the first scan can then be recovered via the ratio PSL_scan1_/PSL_scanN_ = α(1)^β^/αN_unsat_^β^ = 1/N_unsat_^β^_,_ where N_unsat_ is the number of scans required to unsaturate the image plate readout. The fit parameter α cancels in this ratio, and the signal is strictly a function of N_unsat_ and the fit parameter β. Once the total signal was determined from the fit parameter β, the signal was adjusted for any fade that occurred between when the shot was fired and when the image plate was scanned by using the known fade rate formulas given by Boutoux et al.^39^. To convert from PSL to electrons, a conversion factor of 0.026 PSL/electron was used. The calculation of this factor was described in the previous paragraph.

### Error analysis

The largest contribution to the uncertainty in the reported charge is from the variation in β when fitting the decay curves as described in the above paragraph. As stated above, the average β was used to calculate the reported charge. The charge was also calculated using the highest and lowest β value from across the seven fit points. This difference due to the variation in β is included in the error bars for the reported charge. The percent difference in charge by comparing shots taken using different f/#s (f/5, f/6, f/8, and f/10) is ± 16%, and this difference was also included in the error bars in plots when comparing data taken on different f/#s. The error bars also include the small differences in charge due to the uncertainty in the PSL/electron conversion factor itself as reported by Boutoux et al.^39^.

The charge calculation method described has one additional systemic source of uncertainty, which could reduce the overall reported charge by up to a factor of 0.65. As described above, the constant factor used to convert from PSL to electrons was calculated by convolving a representative measured electron spectrum (over the range of 0.9–200 MeV as measured with the EPPS) with the PSL/electron response at each electron energy. The PSL/electron response varies significantly for electron energies below 2 MeV, so uncertainties in the electron spectrum below the lowest measured value of 0.9 MeV could alter the weighted PSL conversion factor and thus the reported charge. To investigate this possibility, the transmission of electrons with energies below 0.9 MeV through the front image plate stack was modelled in Geant4 and convolved with the PSL/electron response in that energy range. The result shows that even if every PSL recorded on the front image plates was due to electrons with energies below 0.9 MeV, which would be the extreme lower bound on the reported charge, the reported charge would only be reduced by a factor of 0.65.

### Conversion efficiency

In order to calculate the conversion efficiency from laser energy to the energy contained in the electron beam, a weighted average electron energy need to be calculated. In the work shown here, this weighted average electron energy was calculated using the representative electron spectrum from this experiment (Fig. 2d). The total charge in the electron beam was integrated by multiplying the signal at each energy by the width of the step in the electron energy in MeV. This integration gives a total number of particles/sr. The electron signal at each electron energy was then divided by the total charge/sr to calculate the fraction of the total charge at each electron energy. The fraction of the total charge at each electron energy can then be multiplied by that energy and summed to get the weighted energy of a typical electron in that spectrum. Once the energy of a typical electron is known, the total charge in that beam can be converted to energy, and from there, the efficiency can be calculated.

## Data Availability

The data that support the plots within this paper and other findings of this study are available from the corresponding author upon reasonable request.
